# Team players against headache: multidisciplinary treatment of primary headaches and medication overuse headache

**DOI:** 10.1007/s10194-011-0364-y

**Published:** 2011-07-21

**Authors:** Charly Gaul, Corine M. Visscher, Rhia Bhola, Marjolijn J. Sorbi, Federica Galli, Annette V. Rasmussen, Rigmor Jensen

**Affiliations:** 1 Headache Centre, Department of Neurology, University Hospital Essen, University Duisburg-Essen, Hufelandstraße 55, 45147 Essen, Germany; 2 Department of Oral Kinesiology, Academic Centre for Dentistry Amsterdam, University of Amsterdam and Free University, Amsterdam, Netherlands; 3 The National Hospital for Neurology and Neurosurgery, London, UK; 4 Department of Clinical and Health Psychology, Utrecht University, Utrecht, Netherlands; 5 IRCCS National Neurological Institute C. Mondino Foundation, Pavia, Italy; 6 Danish Headache Centre, Department of Neurology, Glostrup Hospital, University of Copenhagen, Copenhagen, Denmark

**Keywords:** Multidisciplinary treatment, Headache school, Headache nurse, Physiotherapy

## Abstract

Multidisciplinary approaches are gaining acceptance in headache treatment. However, there is a lack of scientific data about the efficacy of various strategies and their combinations offered by physiotherapists, physicians, psychologists and headache nurses. Therefore, an international platform for more intense collaboration between these professions and between headache centers is needed. Our aims were to establish closer collaboration and an interchange of knowledge between headache care providers and different disciplines. A scientific session focusing on multidisciplinary headache management was organised at The European Headache and Migraine Trust International Congress (EHMTIC) 2010 in Nice. A summary of the contributions and the discussion is presented. It was concluded that effective multidisciplinary headache treatment can reduce headache frequency and burden of disease, as well as the risk for medication overuse headache. The significant value of physiotherapy, education in headache schools, and implementation of strategies of cognitive behavioural therapy was highlighted and the way paved for future studies and international collaboration.

## Introduction

Due to their very high prevalence, headaches cause severe burden of disease on society and high expenses within the health care system [1, 2]. Increasing headache frequency often leads to chronic headache, which is by definition headache on 15 days or more per month for at least 3 months [3]. However, chronic headache is difficult to treat and is very costly. Improved strategies for better prevention and treatment are, therefore, needed. Furthermore, frequent headache also includes the risk of frequent intake of triptans or analgesics resulting in medication overuse headache (MOH), a well-known complication in headache treatment [4, 5]. Patients’ knowledge about headache and its treatment is often poor and may lead to wrong conceptions of disease and insufficient therapy. Likewise, education in non-pharmacological prevention may also be valuable to the patients, preventing chronification and medication overuse. Unfortunately, the possibilities for providing severely affected patients with information and instructions are very limited in many of the settings and countries, mainly due to lack of resources and evidence. Patients in headache centres also stand out in terms of comorbidity and complexity. Thus, medical prophylaxis for headache patients alone very often appears insufficient and additional strategies for these complex patients are urgently needed. Secondary or tertiary care for headache is marked by diversity of required professional expertise. However, cost containment becomes a pertinent challenge as one strives to meet other goals, such as optimal clinical outcome, positive treatment experience and equity in access. An interdisciplinary approach is often recommended and considered to be very highly relevant in providing chronic and often refractory headache patients with appropriate therapeutic care. Establishing a multidisciplinary treatment programme may, therefore, be one step toward optimised care and prevention [6]. According to the European Headache Federation, headache service should be organized in a three-level system reaching from primary headache care to headache clinics and academic headache centres [7]. The major aims of multidisciplinary treatment programmes are to inform and educate patients better in handling headache and to improve therapy in order to reduce headache frequency and enhance quality of life. Multidisciplinary headache programmes have already been established and implemented in several European headache centres, but published outcome data are still sparse.

**Fig. 1:**
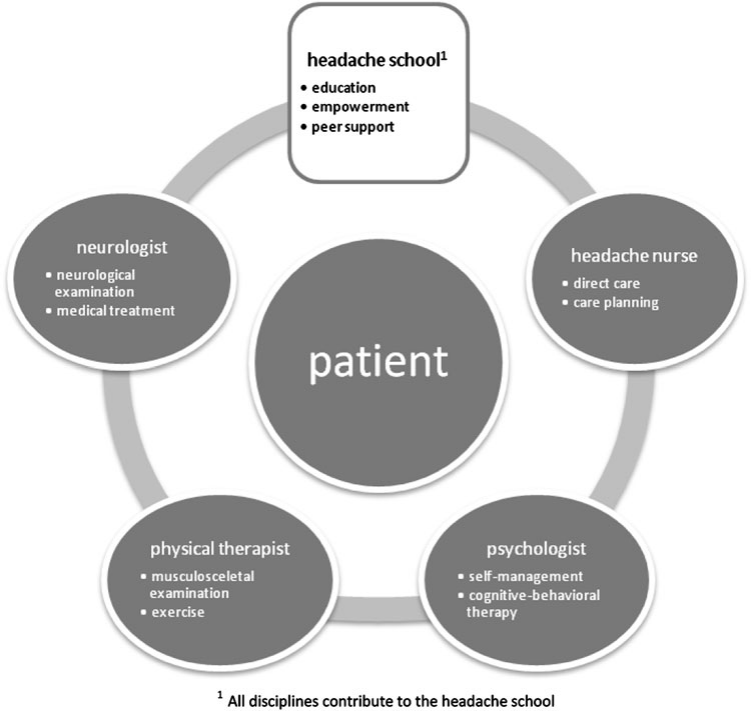
Elements of multidisciplinary approach for complex headache patients

Integrated treatment programmes have been developed and implemented for patients with frequent refractory headaches [8–10]. The concept includes multidisciplinary therapy provided by a team of neurologists, behavioural and clinical psychologists, physical and sports therapists and headache nurses, supplemented by consultants from psychosomatic medicine, psychiatry and dentistry if needed [10] (Fig. 1). To enhance quality of headache treatment, multidisciplinary treatment should be based on teamwork among the different disciplines involved instead of only compiling the concepts of the individual neurologists, psychologists and physical therapists. Which elements of such multidisciplinary approaches are truly relevant and which combinations of treatment strategies should be applied is yet to be established. An overview of published concepts for multidisciplinary treatment is given in Table 1. An important step forward to clarification and closer collaboration between the professions was taken at The European Headache and Migraine Trust International Congress 2010 (EHMTIC 2010) in Nice. An international forum for headache nurses was successfully established by the organization Lifting the Global Burden of Headache (http://www.l-t-b.org) and a specific session on multidisciplinary headache treatment was also organized during EHTMIC 2010. This paper summarises the contributions in this multidisciplinary session and helps pave the way for future collaboration and organizations of the multidisciplinary management of headache.

**Table 1:** Examples of published concepts on multidisciplinary outpatient headache treatment

Centre	Total course (hours)	Involved disciplines				Others
		Neurology	Physical therapy/aerobic endurance sports	Psychology	Headache nurse	
Saskatoon, Canada [62]	6 weeks	X	X	X		Massage therapist, dietician,
Durham, USA [63]	Initial 2 h group visit, no further explanation	X		X		General practitioner
Copenhagen, Denmark [26]	Repeated visits	X	X	X	X	Psychiatrist, dentist, gynecologist
Erlangen, Germany [58]	96-hours (twice weekly 6 h, 8 weeks)	X	X	X		
Copenhagen, Denmark [10]	Group sessions, neurologist; control visits for 20 min every 2-4 months	X	X	X	X	
Alabama, USA [64]	3 × 90 min					Specially-trained migraine patients
Calgary, Canada [65]	Not reported	X	X	X	X	Kinesiologist
Durham, USA [66]	Regular visits, no explanation concerning number and duration	X			X	
Essen, Germany [27]	5 days, 36 h	X	X	X	X	

## The role of the neurologist in the multidisciplinary treatment of headache

The neurologists in a headache center are, and should be, responsible for establishing the correct headache diagnoses according to International Classification of headache Disorders II (ICHD-II) and developing therapy plans in close collaboration with the patients and the team members. A clinical neurological examination should be performed in all patients, supplemented by diagnostic tools if needed to exclude secondary headache [11]. Ideally, the neurologist should then inform the patient about the diagnosis and possible triggering and aggravating factors. Thereafter, an individual plan for acute treatment, and for prophylactic treatment if required, should be developed and explained to the patient. This is usually based on national and international guidelines. Within the multidisciplinary team, the neurologist is the specialist for decisions regarding medical treatment. The neurologist and team members should provide information material about headache disorders and treatment for the patient. In general, neurologists are most accepted by the patient for diagnosis and pharmacological strategies, whereas the planning of the optimal non-pharmacological management needs careful evaluation and collaboration among the entire team and the patient (Fig. 1).

## The role of the physical therapist in the multidisciplinary treatment of headache

The main goal of physical therapy treatments in migraine and tension type headache (TTH) patients is the prevention of headache episodes rather than the alleviation of symptoms once an attack has begun [12–14]. Evidence for the effect of physical therapy is accumulating but the number of high-quality studies is limited and only few evidence-supported treatment recommendations can be made [13, 15]. Neck pain is a very common and prominent symptom in headache patients [16–18] and probably closely related to the pathophysiological mechanisms of many headache disorders. Because of this coexistence with neck pain, physical therapy treatments are often prescribed [17, 19]. Physical therapists are trained to recognise whether disorders of the musculoskeletal system contribute to a patient’s symptoms. This is done by collecting a detailed history from the patient and by conducting a clinical examination. Physical therapy involves a wide range of treatment modalities. Nowadays, active participation of the patient is considered essential for treatment success [16], and the single use of passive interventions (e.g. massage or physical modalities) is no longer considered as ‘best-practice’. There is strong evidence that various types of relaxation training (e.g. thermal/electromyography (EMG) biofeedback) may be an effective treatment for the prevention of migraine [17, 20]. There is weaker evidence that relaxation training may be combined with preventive drug therapy (i.e. propranolol, amitriptyline) to achieve additional improvement in migraine relief and that aerobic exercises are effective in improving quality of life [17, 20]. There is some evidence that the short-term effect of cervical spinal manipulation is comparable to that of amitriptyline for the prevention of migraine [13]. Some evidence for the effectiveness of spinal manipulation towards a reduction of headache episodes has also been presented for TTH [13]. Physical therapists can play an important role in the treatment of patients with secondary headaches and especially those related to a disorder of the musculoskeletal system: ‘headaches attributed to head/neck trauma, cervicogenic headache, or headache or facial pain attributed to a disorder of the temporomandibular joint’ (ICHD-II) [3]. However, the combination of manual therapy and exercise produces greater improvements in pain, function, quality of life and patient satisfaction at both short- and long term [21]. The effects of a therapeutic approach including patients education and physical therapy compared to a control group was investigated in an Italian study suggesting some efficacy [22], while a Finnish cross-over study of physical therapy alone revealed significant benefits on headache and neck pain but not on shoulder symptoms [23]. At present, we can conclude that there is accumulating evidence that physical therapy may be an important element in the multidisciplinary approach, especially in patients with TTH, migraine and cervicogenic headache and headache attributed to a temporomandibular disorder (TMD). Headache patients may benefit from a detailed examination of the cervical spine and the masticatory system to detect possible aggravating factors (in primary headache) or causative factors (in secondary headache). An active treatment strategy including a physical exercise programme may also play an important role in the general health of the patient and in the prevention of headache chronification. However, there’s still a need to investigate the role of physical therapy in the multidisciplinary team and to identify the essential elements of physical therapy.

## The role of headache nurses in the multidisciplinary treatment of headache

In recent decades, the role of the specialist nurse has developed rapidly across specialities but differs across the countries. In general, an international trend is evolving to move nurses’ training into an academic discipline, accompanied by the expansion of nursing research. Integrating nurses into headache care is increasingly regarded as an important step towards improving the outcome for the patients through improved access to services and optimising the consultation time with the neurologist or headache specialist. For example in the UK, where there are currently 12 headache nurses who work within neurology services, a large proportion of patients in these services will have nurse-led care following their initial diagnostic medical consultation. The number of nurses may increase as new services develop and the impact of the nurses’ contribution should be recognised and documented. Similarly, headache specialist nurses are emerging in most other European countries and the newly established International Forum of Headache nurses may, thus, play a role in facilitating service developments, education programmes and delivery across Europe.

It is acknowledged that specialist nurses will go through a role development process to acquire skill and competence to function with maximal effectiveness. Despite the lack of standardised training for headache nurses, it is acknowledged that the best preparation for specialist nursing roles is a combination of having the right experience and suitable educational preparation. Therefore, competence can be established initially through direct patient care, care planning and working with other staff to adapt the care provided for this patient group as appropriate. An initial step in meeting formal education needs of headache nurses was taken in Germany by the headache center in Essen, which provides weekend training courses for headache nurses two times a year. Other upcoming formalised courses will include an online nursing course in the UK delivered through the Migraine Trust.

The specialist nurse may also be involved in conducting or participating in the research and audit as well as planning for changes in patient care delivery based on experience and research. Ultimately, the nursing contribution will become more significant in headache care as services develop and it will probably reflect what is already provided in established multidisciplinary headache clinics [24].

The main activities undertaken by the specialist nurse include patient consultations to monitor their progress at intervals. This includes follow-up to medical clinic consultations or inpatient episodes, monitoring drug efficacy and tolerability, supporting patients with treatment changes and addressing patients’ queries. In many of the settings, specialist nurses will take a headache history, assess level of disability, provide and assess headache diaries and provide support and advice in an outpatient clinic. Typically, the nurse will advise on lifestyle issues, trigger factors, use of medication, change of medication or withdrawal from overused analgesics [24].

In an inpatient hospital setting, the nurse may assess patients’ needs and ensure they understand the plan of care in hospital, monitor progress of treatment, optimise care (and hence resources) and ensure effective discharge planning. Overall, specialist nursing activities are likely to improve both the patient’s experience and clinical organisation because they affect all relevant areas of service delivery. Furthermore, the nurses often participate in research and education to monitor future developments and keep up to date with new and emerging therapies to improve patient and service outcomes [24].

## The role of the headache school in the multidisciplinary headache treatment of headache

The headache school concept may focus on conveying general knowledge to the patients which can be provided by an experienced headache nurse, a psychologist and/or a physician. The lessons can provide knowledge about (a) headache diagnosis (e.g., migraine, tension-type headache, differentiation between the two), (b) attack treatment (c) prophylactic treatment, (d) risk factors and mechanisms which are relevant concerning medication overuse headache and (e) implementation of non-medical prophylactic treatment strategies (endurance sports, relaxation training). The overall goal of the headache school is to qualify the patients themselves as experts for their own headaches. It’s important to assure that the information given in the headache school is compatible with the contents of the whole multidisciplinary treatment programme and according to national treatment guidelines. Therefore, all therapists should know the contents in detail. The information provided to the patients may reduce the risk of delayed recognition of medication overuse and delayed treatment if headaches aggravate. So far, such a concept is not yet evidence-based in headache but similar strategies are widely used and accepted in other chronic disorders, such as ischemic heart diseases, diabetes and stroke [25].

**Fig. 2:**
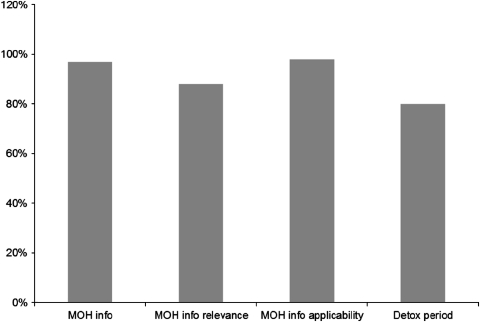
Headache School Copenhagen—patient evaluation (percentage rated as satisfied or very satisfied)

Another concept has been developed at the Danish Headache Center, where 20-25% of patients suffer from medication overuse headache (MOH) [9, 10, 26]. As an essential part of their treatment plan for detoxification, all MOH-patients are offered participation in the Headache School organized as an outpatient class led by nurse specialists. This plan has the aim of supporting and guiding patients during a 2-month period to prevent recurrence of drug overuse. The Headache School in Copenhagen consists of 6 standardised sessions over a period of 3 months, lasting 2 h each. Six to seven patients are seen at each course and a synchronous and abrupt start of the detoxification period is set. During the course, the patients exchange their experiences and hear lectures by nurses, psychologists and physical therapists. Similar concepts in which a headache school is run by physicians, physiotherapists and psychologists are established in other countries [27]. Some examples are given in Table 1. Finally, patients are educated on how to prevent recurrence of drug overuse and MOH. Patients’ satisfaction with the information about MOH was very high and its applicability was reported in more than 90% of the patients and 81% were satisfied or very satisfied with the detoxification and outcome (Fig. 2). In total, the headache frequency and impact on daily life was significantly reduced, so a clear treatment plan and focus on MOH and detoxification were highly recommended [9, 10, 27]. Close follow-up within the headache school concept may also increase motivation. It was concluded that group sessions are recommendable and very suitable with regards to cost-effectiveness and resource demand.

## The role of psychologists in multidisciplinary treatment of headache

Psychiatric comorbidities, like depression or anxiety disorders, are well known and the combination of anxiety and depression was reported in about 20% of the general population in France [28]. Psychiatric comorbidity in general is recognised as a problem in primary care. General practioners reported that those patients are requiring more care, more time, and more frequent consultations [29]. As a consequence of more difficulties to refer such patients to specialists GPs are unsatisfied with the relationship to mental health care providers and ask for better collaboration with them [29]. Therefore, the access for headache patients to psychologists in headache centres is helpful. Transformed migraine seems to have the highest rates of psychiatric comorbidity (78%) compared to chronic tension-type headache (64%) [30]. Involvement of psychiatric disorders was reported for 68% of MOH patients [31, 32]. However, looking at the involvement of psychological factors in headache, we are faced with a lot of different dimensions: from life events to psychological trigger factors, from stress to personality characteristics [33]. As there is a connection between headache and other pain disorders and patients’ psychological health and quality of life, psychologists play an important role in the evaluation of headache patients’ as well as in therapy. A psychological intervention might help to address “modifiable” risk factors for headache chronification [34], such as attack frequency, obesity, medication overuse, stressful life events, caffeine overuse, snoring, and other pain syndromes. Psychological intervention should not only be considered if psychopathology has been diagnosed, but also if psychopathology represents a risk for headache chronification [35]. Furthermore, education and self-management are important to all patients with headache and therefore an important part of the treatment which can be done by psychologists. This includes lifestyle education, self-management, handling medication and risks of medication overuse. Even though detailed scientific data are sparse, psychologists are considered important members of multidisciplinary teams. Non-pharmacological treatments are acknowledged as preventive methods especially for migraine according to neurological guidelines [36]. Psycho-physiological (relaxation often utilised with biofeedback) and cognitive-behavioural training are the core methods of this approach [36–40]. These methods, usually offered in 8–12 (individual) treatment sessions, can be combined, condensed to home-based training [38, 41] and transformed into self-management formats. Such self-management training achieves 42% responders regarding migraine attack prevention (mean change 23%, effect size .6). Furthermore, marked increase in perceived control over and self-confidence in attack prevention and improved migraine-specific quality of life over time were also reported when offered by trained patient trainers supported backstage by a psychologist [42–44]. Essential psychological issues comprise self-efficacy, perceived control and catastrophizing, and the patient’s readiness to change [40] and avoidance [41] should be considered. Self-efficacy mediates successful headache management and is related to perceived own control over headache [42–47]. Catastrophizing, on the other hand, is associated with reduced functioning and quality of life in severe migraine [48, 49] and with more pain and disability in chronic pain [50, 51]. The focus should be not only on the avoidance of headache triggers, but the therapy also working on active management and coping of headche [45].

The psychological work may be enhanced by the aid of testing evaluating psychiatric disorders (e.g. Mini Psychiatric Interview), personality (e.g. Shedler–Westen Assessment procedure) and cognitive factors as locus of control and self-efficacy (e.g. Headache specific locus of control (LOC) Scale, Headache Management Self-Efficacy Scale). New aspects are the internet-based protocols for cognitive-behavioural self-management, guided training and treatment to be utilised as part of primary care, intermediate care and self-care, which are currently under the development and evaluation in the Netherlands [52]. Of the three early attempts to utilise the internet for the purpose of self-help and behavioural management in primary headache, two suffered from a lack of diagnostic specificity and methodological limitations [53, 54]. The best-designed study [55] involved 156 participants with a reported medical diagnosis of either migraine or TTH in a randomised controlled trial with promising results but a drop-out rate of over 40%.

## Discussion

As it has been documented that medical prophylaxis for headache patients, especially migraine, alone is only effective in about half of the headache patients, additional strategies are urgently needed [56]. Behavioural therapy alone is not more effective, but the combination of the two is superior to the single therapies [57]. To provide an appropriate therapeutic concept to patients with chronic and difficult to treat headaches, an interdisciplinary approach is often recommended and is considered to be very relevant. However, the ideal duration and setting for such a multidisciplinary treatment programme are still under debate [58]. It has been proposed that there is at least a need for improving education of patients about lifestyle changes and non-pharmacological-based therapy approaches. Furthermore, education about acute and prophylactic treatment is needed, since patient empowerment may improve adherence and compliance with treatment recommendations, which is quite insufficient in many of the patients. Recently, it has been shown that adherence to non-pharmacological treatment recommendations was associated with better outcome and reduction of headache days per month in a multidisciplinary treatment programme [27].

The main difficulties in outcome evaluation of multidisciplinary treatment concepts are: (a) measurement of overall outcome (for example headache days and quality of live) does not allow conclusions about the efficacy of the implement different parts of such a modular treatment concept. (b)Comparing a standard treatment with multidisciplinary treatment is very difficult, because a lack of equally affected patients (regarding number of headache days, psychiatric comorbidity, and burden of disease) in the headache centres not undergoing the same treatment concept. The optimal evaluation will be done in a randomised trial, comparing multidisciplinary treatment with a placebo condition, which is not available for multidisciplinary treatment. (c) Observational studies in the headache centers may be influenced by different treatment motivation of patients participating in multidisciplinary treatment compared to patients not participating.

International collaboration of headache centres may also establish and intensify interdisciplinary contacts and research, resulting in improved treatment in the future. Despite different conditions within various national healthcare systems, headache therapists may learn from each other and develop new and more effective strategies in interdisciplinary treatment. Internet-based instruments, such as electronic headache diaries, can be optimized, tested and implemented in clinical trials and thereby be used in different countries and languages for early and effective implementation of promising new strategies. More intensive exchange of research findings and experiences in the treatment between academic as well as non-academic headache treatment providers may also result in synergisms, better treatment options and higher job satisfaction of the members of the staff in headache centres.

Headache patients with more complex problems in the terms of potentially modifiable risk factors [6], such as substantial stressful life events, sleep problems or emerging anxiety or depression, are in need of help provided by psychologists with expertise in headache disorders. Multidisciplinary headache centres operating in the second or tertiary level of health care should work in close collaboration with regional health care institutions as well as with consultants with specific interest or experience in such complicated and refractory chronic pain patients.

Future activities should consider new technologies like internet or smart phones for patients’ self-management or online diaries. These instruments could be further established and tested in the prospective studies [59–61]. Internet-based training, online migraine monitoring and methods for mobile monitoring and coaching are promising tools to increase the outreach of behavioural support and psychological guidance in the field of primary headache. However, this has to be done in a way that is both cost-efficient and highly accepted by headache patients as well as headache centres.

In conclusion, there is a strong need for evaluation, closer collaboration and more research on multidisciplinary treatment. Furthermore, it is also important to identify which contingent of different modalities should be part of a multidisciplinary treatment programme for the individual patient. Even though medical therapy alone may be fully appropriate in patients with infrequent headache without comorbidities, a multidisciplinary approach is recommendable in more complicated and severely affected patients. Considering the high manpower requirements of a multidisciplinary team we suggest further socioeconomic analysis of these concepts to prove cost efficacy of the treatment with respect to quality of life, direct and indirect costs.

## References

[1] Hu XH, Markson LE, Lipton RB, Stewart WF, Berger ML (1999). Burden of migraine in the United States: disability and economic costs. Arch Intern Med.

[2] Munakata J, Hazard E, Serrano D, Klingman D, Rupnow MF, Tierce J (2009). Economic burden of transformed migraine: results from the American Migraine Prevalence and Prevention (AMPP) Study. Headache.

[3] InternationalHeadache Society (2004). The international classification of headache disorders: second edition. Cephalalgia.

[4] Diener HC, Limmroth V (2004). Medication-overuse headache: a worldwide problem. Lancet Neurol.

[5] Evers S, Marziniak M (2010). Clinical features, pathophysiology, and treatment of medication-overuse headache. Lancet Neurol.

[6] Scher AI, Midgette LA, Lipton RB (2008). The chronification of headache. Headache.

[7] Steiner TJ, Antonaci F, Jensen R, Lainez MJ, Lanteri-Minet M, Valade D (2011) Recommendations for headache service organisation and delivery in Europe. J Headache Pain. doi:10.1007/s10194-011-0320-x 10.1007/s10194-011-0320-x PMC3139057 21380555

[8] Antonaci F, Valade D, Lanteri-Minet M, Láinez JM, Jensen R, Steiner TJ, European Headache Federation and Lifting The Burden: the Global Campaign to Reduce the Burden of Headache Worldwide (2008). Proposals for the organisation of headache services in Europe. Intern Emerg Med.

[9] Zeeberg P, Olesen J, Jensen R (2006). Probable medication-overuse headache: the effect of a 2-month drug-free period. Neurology.

[10] Jensen R, Zeeberg P, Dehlendorf C, Olesen J (2010). Predictors of outcome of a multidisciplinary programme in a multidisciplinary headache center. Cephalalgia.

[11] Dodick DW (2010). Pearls: headache. Semin Neurol.

[12] Campbell JK, Penzien DB and Wall EM (2010) Evidenced-based guidelines for migraine headache: behavioral and physical treatments. https://www.americanheadachesociety.org/professionalresources/USHeadacheConsortiumGuidelines.asp

[13] Bronfort G, Nilsson N, Haas M, Evans R, Goldsmith CH, Assendelft WJ et al. (2004) Non-invasive physical treatments for chronic/recurrent headache. Cochrane Database Syst Rev CD001878 10.1002/14651858.CD001878.pub2 15266458

[14] Torelli P, Jensen R, Olesen J (2004). Physiotherapy for tension-type headache: a controlled study. Cephalalgia.

[15] Gaul C, Busch V (2009). Impact of physiotherapy, massages and lymphatic drainage in migraine therapy. Schmerz.

[16] Bendtsen L, Jensen R (2009). Tension-type headache. Neurol Clin.

[17] Biondi DM (2005). Physical treatments for headache: a structured review. Headache.

[18] Blau JN, MacGregor EA (1994). Migraine and the neck. Headache.

[19] Gaul C, Eismann R, Schmidt T, May A, Leinisch E, Wieser T (2009). Use of complementary and alternative medicine in patients suffering from primary headache disorders. Cephalalgia.

[20] Silberstein SD (2000). Practice parameter: evidence-based guidelines for migraine headache (an evidence-based review): report of the Quality Standards Subcommittee of the American Academy of Neurology. Neurology.

[21] Miller J, Gross A, D’Sylva J, Burnie SJ, Goldsmith CH, Graham N (2010). Manual therapy and exercise for neck pain: a systematic review. Man Ther.

[22] Mongini F, Ciccone G, Rota E, Ferrero L, Ugolini A, Evangelista A (2008). Effectiveness of an educational and physical programme in reducing headache, neck and shoulder pain: a workplace controlled trial. Cephalalgia.

[23] Sjogren T, Nissinen KJ, Jarvenpaa SK, Ojanen MT, Vanharanta H, Malkia EA (2005). Effects of a workplace physical exercise intervention on the intensity of headache and neck and shoulder symptoms and upper extremity muscular strength of office workers: a cluster randomized controlled cross-over trial. Pain.

[24] Bhola R, Goadsby PJ (2011). A trans-cultural comparison of the organisation of care at headache centres world-wide. Cephalalgia.

[25] Rajpathak SN, Aggarwal V, Hu FB (2010). Multifactorial intervention to reduce cardiovascular events in type 2 diabetes. Curr Diab Rep.

[26] Zeeberg P, Olesen J, Jensen R (2005). Efficacy of multidisciplinary treatment in a tertiary referral Headache Center. Cephalalgia.

[27] Gaul C, van Doorn C, Webering N, Dlugaj M, Katsarava Z, Diener HC et al. (2011) Efficacy of a headache specific multidisciplinary treatment program and adherence to treatment in a tertiary headache center. J Headache Pain. doi:10.1007/s10194-011-0348-y 10.1007/s10194-011-0348-y PMC3139052 21544647

[28] Lantéri-Minet M, Radat F, Chautard MH, Lucas C (2005). Anxiety and depression associated with migraine: influence on migraine subjects’ disability and quality of life, and acute migraine management. Pain.

[29] Younes N, Gasquet I, Gaudebout P, Chaillet MP, Kovess V, Falissard B (2005). General practitioners’ opinions on their practice in mental health and their collaboration with mental health professionals. BMC Fam Pract.

[30] Juang KD, Wang SJ, Fuh JL, Su TP (2000). Comorbidity of depressive and anxiety disorders in chronic daily headache and its subtypes. Headache.

[31] Puca F, Genco S, Prudenzano MP, Savarese M, Bussone G, D’Amico D (1999). Psychiatric comorbidity and psychosocial stress in patients with tension-type headache from headache centers in Italy. The Italian Collaborative Group for the Study of Psychopathological Factors in Primary Headaches. Cephalalgia.

[32] Atasoy HT, Atasoy N, Unal AE, Emre U, Sumer M (2005). Psychiatric comorbidity in medication overuse headache patients with pre-existing headache type of episodic tension-type headache. Eur J Pain.

[33] Guidetti V, Galli F, Sheftell F (2010). Headache attributed to psychiatric disorders. Handb Clin Neurol.

[34] Bigal ME, Lipton RB (2009). What predicts the change from episodic to chronic migraine?. Curr Op Neurol.

[35] Guidetti V, Galli F, Fabrizi P, Napoli L, Giannantoni AS, Bruni O (1998). Headache and psychiatric comorbidity: clinical aspects and outcome in an 8-year follow-up study. Cephalalgia.

[36] Antonaci F, Dumitrache C, De Cillis I, Allena M (2010). A review of current European treatment guidelines for migraine. J Headache Pain.

[37] Grazzi l (2007). Behavioral treatments: rationale and overview of the most common therapeutic protocols. Neurol Sci.

[38] Andrasik F (2007). What does the evidence show? Efficacy of behavioral treatments for recurrent headaches in adults. Neurol Sci.

[39] Buse DC, Andrasik F (2009). Behavioral medicine for migraine. Neurol Clin.

[40] Nicholson R (2010). Chronic headache: the role of the psychologist. Curr Pain Headache Rep.

[41] Haddock CK, Rowan AB, Andrasik F, Wilson PG, Talcott GW, Stein RJ (1997). Home-based behavioural treatments for chronic benign headache: a meta-analysis of controlled trials. Cephalalgia.

[42] Mérelle SYM, Sorbi MJ, van Doornen LJP, Passchier J (2008). Migraine patients as trainers of their fellow patients in non-pharrmacological preventive attack management: short-term effects of a randomized controlled trial. Cephalalgia.

[43] Mérelle SYM, Sorbi MJ, van Doornen LJP, Passchier J (2008). Lay trainers with migraine for a home-based behavioural training: a 6-month follow-up study. Headache.

[44] Mérelle SYM, Sorbi MJ, Duivenvoorden HJ, Passchier J (2010). Qualities and health of lay trainers with migraine for behavioural attack prevention. Headache.

[45] Martin PR (2010). Managing headache triggers: think’ coping’ not’ avoidance’. Cephalalgia.

[46] Holroyd KA, Labus JS, Carlson B (2009). Moderation and mediation in the psychological and drug treatment of chronic tension-type headache: the role of severity and psychiatric comorbidity. Pain.

[47] Hansen JS, Bendtsen L, Jensen R (2009). Psychometyric properties of the Danish versions of Headache-Specific Locus of control Scale and Headache Management Self-Efficacy Scale. J Headache Pain.

[48] Holroyd KA, Drew JB, Cottrell CK, Romanek KM, Heh V (2007). Impaired functioning and quality of life in severe migraine: the role of catastrophizing and associated symptoms. Cephalalgia.

[49] Seng EK, Magyar A, Holroyd KA, Drew JB, Cottrel CC (2010). Decreases in catastrophizing are associated with decreases in disability over behavioral migraine treatment. Int J Behav Med.

[50] Sorbi MJ, Peters ML, Kruise DA, Maas CJM, Kerssens JJ, Verhaak PFM (2006). Electronic momentary assessment in chronic pain I: psychological pain responses as predictors of pain intensity. Clin J Pain.

[51] Sorbi MJ, Peters ML, Kruise DA, Maas CJM, Kerssens JJ, Verhaak PFM (2006). Electronic momentary assessment in chronic pain II: pain and psychological pain responses as predictors of pain disability. Clin J Pain.

[52] Sorbi MJ, van der Vaart R (2010). User acceptance of an internet training aid for migraine self-management. J Telemed Telecare.

[53] Strom L, Peterson R, Andersson GA (2000). A controlled trial of self-help treatment of recurrent headache conducted via the internet. J Consult Clin Psychol.

[54] Andersson G, Lundstrom P, Strom L (2003). Internet-based treatment of headache: does telephone contact add anything?. Headache.

[55] Devineni T, Blanchard EB (2005). A randomized controlled trial of an internet-based self-help treatment for chronic headache. Behav Res Ther.

[56] Goadsby PJ, Sprenger T (2010). Current practice and future directions in the prevention and acute management of migraine. Lancet Neurol.

[57] Holroyd KA, Cottrell CK, O’Donnell FJ, Cordingley GE, Drew JB, Carlson BW (2010). Effect of preventive (beta blocker) treatment, behavioural migraine management, or their combination on outcomes of optimised acute treatment in frequent migraine: randomised controlled trial. BMJ.

[58] Gunreben-Stempfle B, Griessinger N, Lang E, Muehlhans B, Sittl R, Ulrich K (2009). Effectiveness of an intensive multidisciplinary headache treatment program. Headache.

[59] Kleiboer AM, Sorbi MJ, Mérelle SYM, Passchier J, van Doornen LJP (2009). Utility and preliminary effects ‘of ‘online digital assistance’ for behavioural attack prevention in chronic migraine. Telemed J e-Health.

[60] Sorbi MJ, Mak SB, Houtveen J, Kleiboer AM, van Doornen LJP (2007). Mobile web-based monitoring and coaching: feasibility in chronic migraine. J Med Internet Res.

[61] Munksgaard SB, Allena M, Tassorelli C, Rossi P, Katsarava Z, Bendtsen L (2011). The Comoestas Consortium. What do the patients with medication overuse headache expect from treatment and what are the preferred sources of information?. J Headache Pain.

[62] Lemstra M, Stewart B, Olszynski P (2002). Effectiveness of multidisciplinary intervention in the treatment of migraine: a randomized clinical trial. Headache.

[63] Harpole LH, Samsa GP, Jurgelski AE, Shipley JL, Bernstein A, Matchar DB (2003). Headache management program improves outcome for chronic headache. Headache.

[64] Rothrock JF, Parada VA, Sims C, Key K, Walters NS, Zweifler RM (2006). The impact of intensive patient education on clinical outcome in a clinic-based migraine population. Headache.

[65] Magnusson JE, Riess CM, Becker WJ (2004). Effectiveness of a multidisciplinary treatment program for chronic daily headache. Can J Neurol Sci.

[66] Matchar DB, Harpole L, Samsa GP, Jurgelski A, Lipton RB, Silberstein SD (2008). The headache management trial: a randomized study of coordinated care. Headache.

